# Development of a double-recombinant antibody sandwich ELISA for quantitative detection of epsilon toxoid concentration in inactivated *Clostridium perfringens* vaccines

**DOI:** 10.1186/s12917-020-02572-4

**Published:** 2020-09-29

**Authors:** Maryam Alibeiki, Mehdi Golchin, Mohammad Tabatabaei

**Affiliations:** 1 Department of Pathobiology, Faculty of Veterinary Medicine, Shiraz University, Shiraz, Iran; 2 Department of Pathobiology, Faculty of Veterinary Medicine, Shahid Bahonar University of Kerman, Kerman, Iran

**Keywords:** Epsilon toxin, *Clostridium perfringens*, Recombinant antibody, Phage display, Sandwich ELISA

## Abstract

**Background:**

Epsilon toxin (ETX) causes a commonly fatal enterotoxemia in domestic animals. Also, ETX causes serious economic losses to animal husbandry. In this study, we selected several clones against ETX using repertoires displayed on filamentous phage. Anti-ETX specific clones were enriched by binding to immobilized antigen, followed by elution and re-propagation of phage. After multiple rounds of binding selection, ELISA analysis showed that most isolated clones had high affinity and specificity for ETX.

**Results:**

Two recombinant monoclonal antibodies against ETX were isolated by phage display technology. B_1_ phage VH antibody isolated from DAb library and G_2_ soluble scFv antibody isolated from Tomlinson I + J libraries have been applied as the capture and detection antibodies for developing an ETX sandwich ELISA test, respectively.

**Conclusions:**

Designed ETX sandwich ELISA could be a valuable tool for quantitative detection of ETX in inactivated commercial vaccines against enterotoxemia.

**Graphical abstract:**

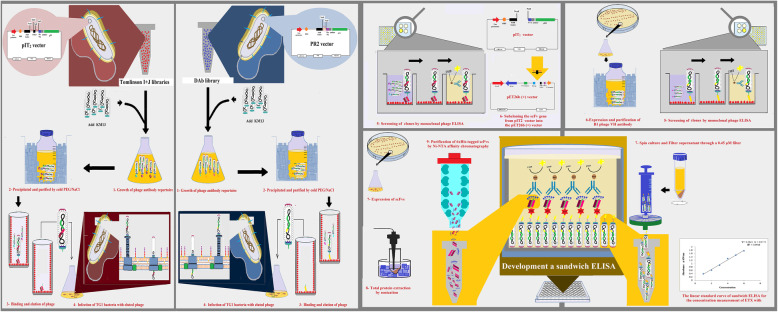

## Background

Epsilon toxin (ETX) is a 33 kDa protein and one of the pore-forming toxins synthesized by *Clostridium perfringens* type B and D strains [1]. ETX has high potency and the Centers for Disease Control and Prevention (CDC) categorized epsilon toxin as the second highest priority agents (Category B) [2]. This toxin plays a significant role in causing enterotoxemia in domestic ruminants, especially sheep, and can cause sudden death and severe economic losses [3]. Enterotoxemia begins when *C. perfringens* type B or D strains secrete ETX prototoxin into the intestinal lumen [4]. At first, the toxin is inactive, but it is activated after cleaving the 13 N-terminal and 22 C-terminal residues by proteases [5]. After activation, ETX forms the pores across the cell membrane of ruminant cells and alters the permeability of cell monolayers, such as epithelium and endothelium, and leads to necrotic lesions and perivascular edema in different tissues, especially in kidney and brain cells [6, 7]. An effective manner to control ETX-induced enterotoxemia in domestic ruminants is vaccination. Commercially available vaccines are based on inactivated toxins isolated from formaldehyde-treated bacterial culture filtrate. Unfortunately, these vaccines usually have many production defects and have variable immunogenicity. One of these defects is the non-uniform concentration of antigens in each batch that leads to potency variation from batch to batch [8–11]. Therefore, developing new methods for the detection and quantitative measurement of antigens in each batch of the vaccines is required. One of the most appropriate methods for quantitative detection of a specific protein in a complex mixture is sandwich Enzyme-Linked Immunosorbent Assay (ELISA) that is used as a common tool for clinical and research programs. As a rapid analysis and inexpensive assay, ELISA has many benefits like sensitivity, specificity, simplicity, and stability [12–14].

The aim of this study was to isolate specific recombinant antibody fragments from Human Domain Antibody (DAb) and Tomlinson I + J libraries as capture and detector antibodies for developing a sandwich ELISA for quantitative detection of ETX in inactivated commercial enterotoxemia vaccines.

## Results

### Biopanning of phage display libraries

At first, the molecular size and purity of the epsilon toxoid were confirmed by SDS-PAGE. Then to isolate specific phage antibodies against ETX, two selections in three rounds were performed using the Tomlinson I + J and DAb libraries. In each round of selection, the phages with a binding affinity for ETX were isolated. After amplification, the phages were used for the next round of selection.

### Screening of clones by polyclonal and monoclonal phage ELISA

**Fig. 1:**
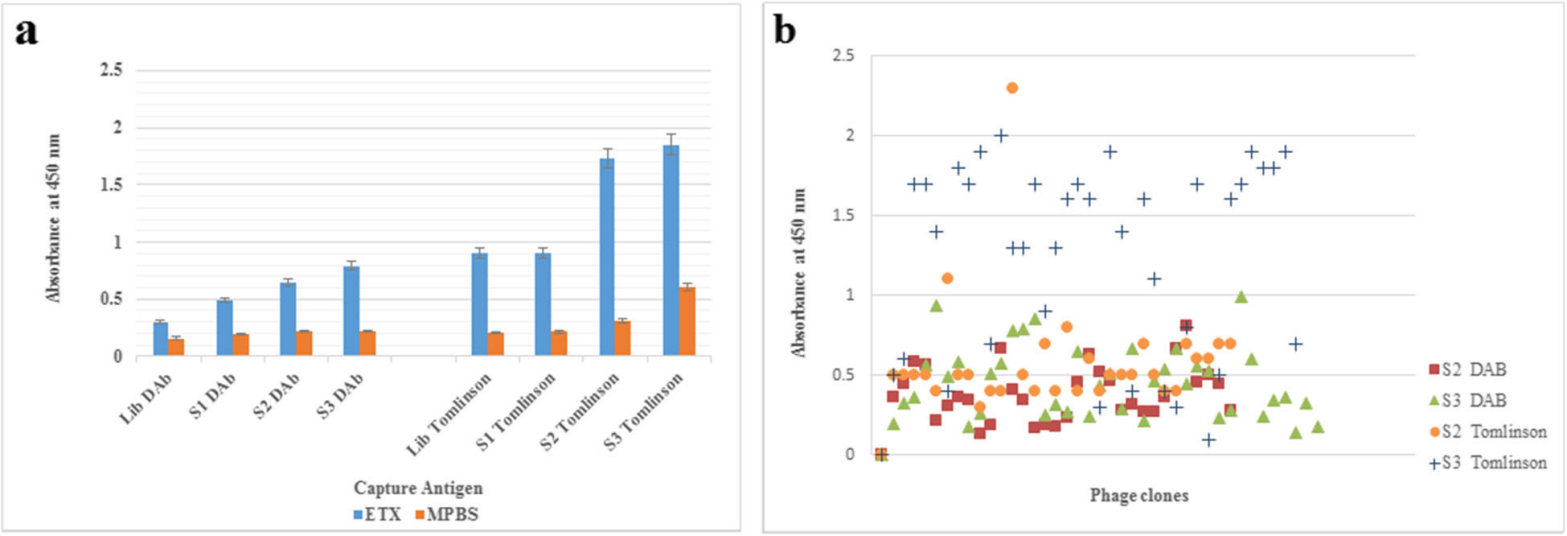
**a**. Screening of clones by polyclonal phage ELISA. Epsilon toxoid was coated to plastic and then detected with an anti-c-Myc antibody. The coating was carried out in duplicate; the mean value is presented, the error bars indicat the standard deviation of the two values. 5% MPBS buffer was coated as a negative control. S_1_, S_2_, S_3_ = selection rounds 1, 2 and 3. **b**. Screening of clones by monoclonal phage ELISA of Tomlinson I + J and DAb libraries. Individual phage clones from the second and third rounds of selection were tested by monoclonal phage ELISA against purified epsilon toxoid. Phages were applied as follows; S_2_: 32 clones picked at random after round 2 of selection; S_3_: 36 clones picked at random after round 3 of selection. OD at 450 nm was measured after 10 minutes.

The populations of phage recovered after each round of selection from each library were screened against ETX by polyclonal phage ELISA. The results of polyclonal ELISA tests showed that the reactivity towards the epsilon toxoid increased from the first round to third round, respectively. These results indicated that phage antibodies reactive with epsilon toxoid were successfully isolated and enriched from both libraries (Fig. 1a ).

Also, phages were prepared individually from clones of second and third rounds of selection from each library (Tomlinson I + J and DAb) for analysis by monoclonal phage ELISA. As Fig. 1b shows, most of the isolated clones, especially from Tomlinson I + J libraries had a high affinity toward antigen.

In the next steps, the best-isolated clone from monoclonal scFv ELISA of Tomlinson I + J libraries (Clone No. G_2_) was used for expression soluble scFvs, and the best-isolated phage from DAb Library (Clone No. B_1_) was used for designing sandwich ELISA.

### Expression and purification of G_2_ scFv

To improve the expression of recombinant anti-ETX G_2_ scFv, the nucleotide sequence of the gene encoding anti-ETX G_2_ scFv was subcloned into the pET26b(+) vector using *Not*I and *Nco*I restriction enzymes. Following subcloning and transformation, the presence of the full-length insert of a subcloned gene was analyzed by double digestion using the above restriction enzymes.

After expression, soluble G_2_ scFv was purified by nickel affinity chromatography and then analyzed by SDS-PAGE and Western blotting using HRP-conjugated monoclonal anti-polyhistidine antibody. The SDS-PAGE analysis confirmed the successful expression of the gene G_2_ scFv in *E. coli* BL21 (DE3) and also the presence of soluble G_2_ scFv after extraction and purification steps. The Western blot result confirmed G_2_ scFv antibody recognition.

### Development of an ETX sandwich ELISA for quantitative detection of ETX

**Fig. 2:**
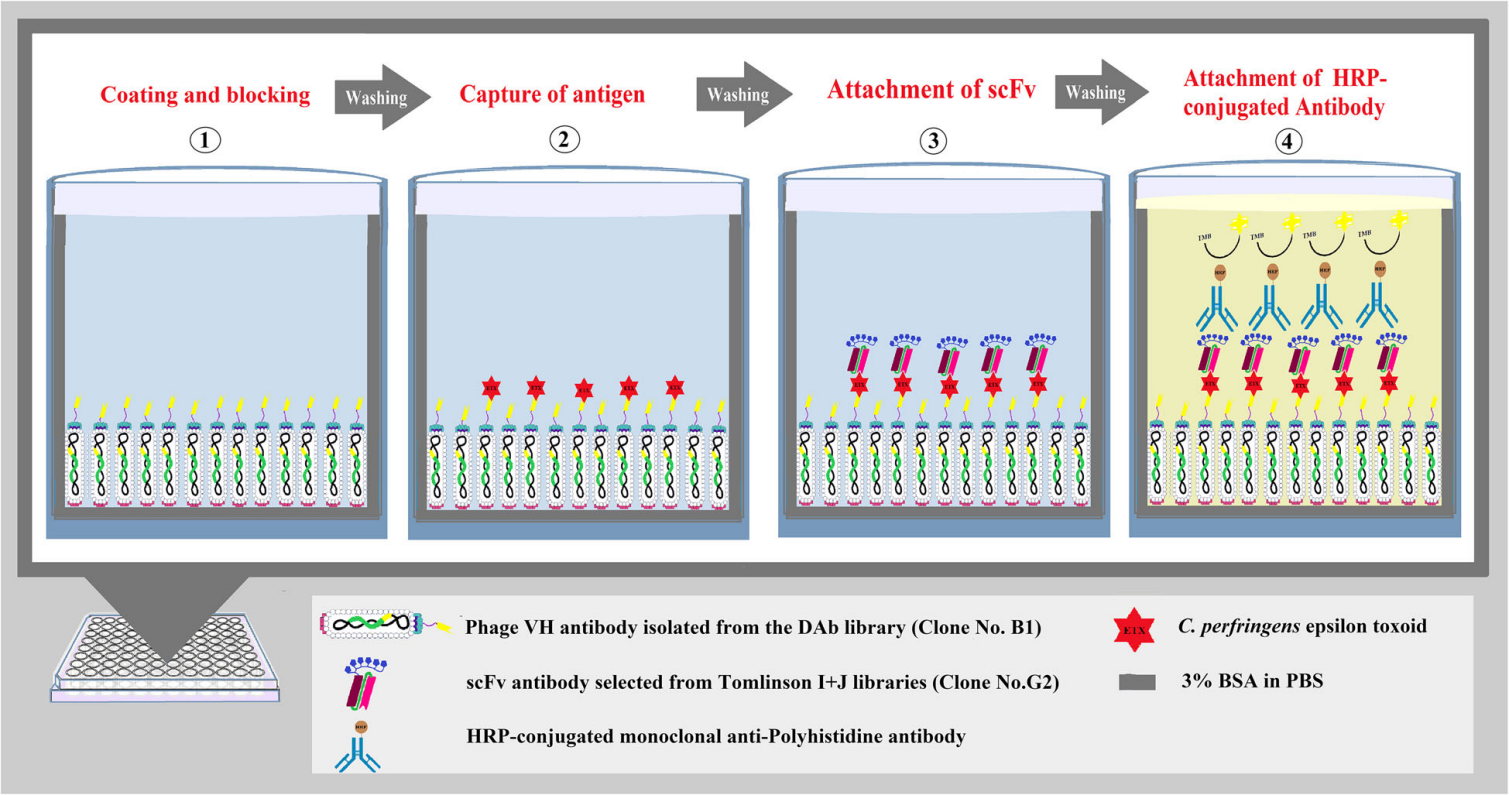
Schematic illustrations of double-recombinant antibody sandwich ELISA for quantitative measurement of ETX. (1) Well is coated with the B_1_ phage VH antibody isolated from the DAb library and the uncoated surface is blocked by 3% BSA/PBS. (2) The *C. perfringens* epsilon toxoid binds to the coated B_1_ phage VH antibody. (3) G_2_ soluble scFv antibody selected from Tomlinson I + J libraries as the detector antibody binds to epsilon toxoid. (4) HRP-conjugated monoclonal anti-Polyhistidine antibody as the conjugate antibody binds to hexahistidine tag fused to G_2_ scFv

**Fig. 3:**
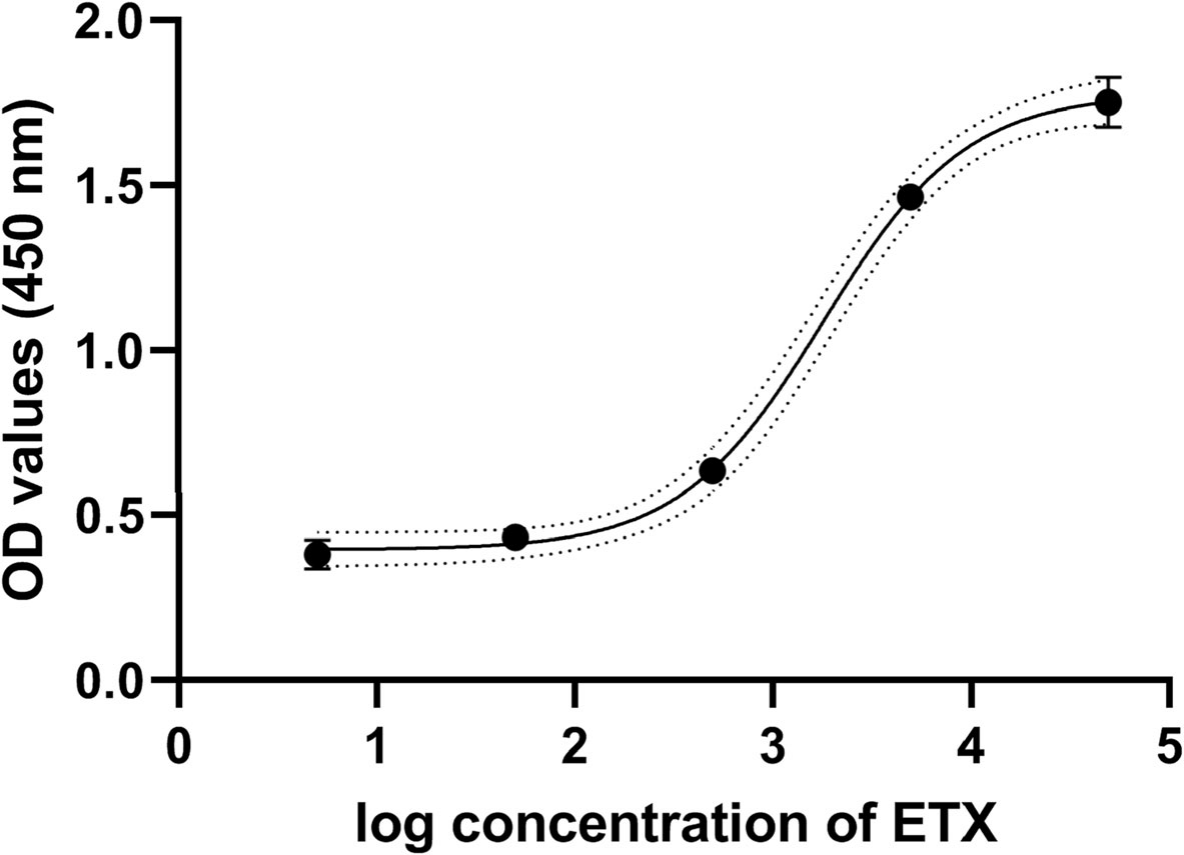
The standard curve of the double-recombinant antibody sandwich ELISA for epsilon toxoid with *R*^2^ = 0/997. Each point is the mean ± standard deviation (*n* = 2)

An ETX sandwich ELISA test was designed to determine the concentration of ETX using two recombinant antibodies isolated from the DAb and Tomlinson I + J libraries as shown in Fig. 2. The results of titration showed that the optimal concentration of the capture B_1_ phage VH antibody was 25 µg/ml (100 µl), and the detector G_2_ scFv antibody was 50 µg/ml (100 µl). Also, 1:5000 HRP-conjugated anti-his-tag antibody was used as the conjugated antibody. The standard curve of the double-recombinant antibody sandwich ELISA for epsilon toxoid with *R*^2^ = 0/997 was calculated using GraphPad Prism 8 software and 4- parameter logistic curve (4pl) to fit the standard curve (Fig. 3). The limits of detection (LOD), limits of quantification (LOQ) were calculated by the standard formula as mentioned in the Methods section. LoD and LoQ were 5.37 ng/ml and 115.36 ng/ml, respectively. The approximate detection range was about 120 ng/ml to 50,000 ng/ml. The CV% of standards and a vaccine sample concentration are presented in Table 1.

**Table 1:** A vaccine sample concentration (ng/ml) obtained from calibration curve

Optical density (450 nm)					Vaccine sample conc (ng/ml)			
Std Conc (ng/ml)	Values	Mean	SD	CV%	Values	Mean	SD	CV%
5	0.413 0.352	0.3825	0.043134	11.31	2039 2350	2194	219	10
50	0.441 0.426	0.4335	0.010607	2.44				
500	0.638 0.635	0.6365	0.002121	0.31				
5000	1.459 1.471	1.465	0.008485	0.54				
50,000	1.806 1.699	1.7525	0.07566	4.28				

Standard curve was based on five serial dilutions of ETX with a concentration range of 5 to 50,000 ng/ml.

CV%: Coefficient of variation expressed as percentage; *Std conc* Standard concentration; *SD* Standard deviation.

## Discussion

Previous studies reported many problems with commercial enterotoxemia vaccines, such as the difference in antigen concentration and potency of the vaccine from batch to batch which cause inequality of antigen concentration distributed in each injection vial [8, 11, 15, 16] so that potency of the vaccine varies from batch to batch [9, 10, 17]. In order to detect and measure the *C. perfringens* epsilon toxoid in each batch, and evaluatethe immunogenicity of vaccines prepared, we developed an ETX sandwich ELISA test using specific recombinant antibodies against pure *C. perfringens* epsilon toxoid. We used recombinant antibodies because recombinant monoclonal antibody fragments have several advantages in comparison with conventional monoclonal antibodies and have been proposed as alternative tools for various diagnostic purposes [18, 19]. These proteins can be easily and economically produced in various expression systems, for example, *E. coli*, plant, yeast, and cell culture and their biological activities can be readily improved by *in vitro* techniques [19, 20].

To isolate specific antibodies against the important epsilon toxoid, we used the two DAb and Tomlinson I + J phage display libraries. These libraries have billions of different antibody fragments built simultaneously with high expression, proper folding and low toxicity for *E. coli* cells. After three rounds of selection, and evaluation by polyclonal and monoclonal phage ELISA, several clones from rounds two and three showed high reactivity against ETX toxoid. Nine of the best clones were chosen for further experiments. Finally, clone B_1_ from DAb library for producing anti-ETX phage antibody and clone G_2_ from Tomlinson I + J libraries were used for making anti-ETX scFv to design the ETX sandwich ELISA.

As previously indicated, the double antibody sandwich ELISA has higher sensitivity and specificity than indirect ELISA, which can accurately quantify antigens with simple operation [21–24]. We designed a sandwich ELISA for the measurement of the *C. perfringens* epsilon toxoid. Both purified anti-ETX scFv and purified anti-ETX phage antibody were evaluated as a capture antibody for coating microtiter plates or as detector antibody, and their affinity and efficiency toward antigen were checked. Then, the coated plates were tested for dose-dependent reactions against a dilution series of ETX.

The results showed our phage antibody fragment can capture antigen much better than scFv antibody (data not shown). Then, the optimum concentration of both recombinant anti-ETX antibodies were applied for testing dose-dependent reactions against a dilution series of ETX and an inactivated enterotoxemia commercial vaccine (Razi Vaccine and Serum Research Institute (RVSRI)). Fortunately, the designed sandwich ELISA test was able to determine the concentration of ETX in the commercial vaccine.

In summary, we isolated two recombinant monoclonal antibodies (B_1_ and G_2_) with a high affinity to ETX from two synthetic libraries by phage display technology. We established a sandwich ELISA using the B_1_ phage antibody for capture and G_2_ scFv antibody fragment as a detector of ETX. This sandwich ELISA could be used for quick detection of ETX and monitoring vaccine quality of commercial enterotoxaemia vaccines.

## Conclusions

The results of this study show that the designed ETX sandwich ELISA is a rapid, sensitive, and reliable method for quantitative detection of ETX and assessment of ETX in commercial vaccines against enterotoxemia.

## Methods

The Tomlinson I + J human single fold synthetic naïve phage display single-chain antibody fragment (scFv) libraries constructed in pIT_2_ (HIS MYC tag) vector with the size of 1.4 × 10^8^ for each, and DAb human VH domain library constructed in PR_2_ (MYC VSV tag) with the size of 3 × 10^9^, helper phage KM13, *E. coli* strains TG_1_ and HB_2151_ for selection of specific antibody clones and production of phage and soluble single-chain Fvs antibodies, respectively, were purchased from GeneService (Cambridge, UK). *C. perfringens* epsilon toxoid was purchased from National Institute for Biological Standards and Control (NIBSC), UK.

### Biopanning of phage display libraries

The purity of the epsilon toxoid was initially confirmed by SDS-PAGE. The libraries and KM13 helper phage stocks were amplified to have enough quantities for use in several rounds of selection. Biopanning was performed using Tomlinson I + J and DAb libraries in parallel to ensure the researchers select the most epsilon toxoid binding clones. For selection, MaxiSorp® immunotubes (Nunc, Denmark) were coated with 5 ml pure *C. perfringens* epsilon toxoid (100 µg/ml) in carbonate buffer (pH 9.4) and incubated overnight at 4 °C, washed three times with PBS and blocked with 4% MPBS (4% Dry skimmed milk in PBS) buffer overnight at 4 °C. The next day, 5 × 10^12^ purified phages from each library in 5 ml MPBS buffer were added to each immunotube and incubated for one hour with gentle agitation at room temperature. The unbound phages were removed by washing 10 times with PBS containing 0.1% Tween 20 and twice with PBS buffer. To recover bound phages, 5 ml trypsin solution (1 mg/10 ml) was added to each immunotube and incubated for one hour at room temperature with gentle agitation. Then, eluted phages (5 ml) were used to infect exponentially growing *E. coli* TG_1_ (30 ml), incubated at 37 °C for one hour in a water bath and plated onto TYE agar plates (100 µg/ml ampicillin, 4% glucose) and incubated at 30 °C overnight. The following day, cells were scraped from agar plates and diluted into 500 ml of 2xTY medium (100 µg/ml ampicillin, 4% glucose) and infected with KM13 phage. Then, grown phages were precipitated and purified by cold PEG/NaCl solution (20% Polyethylene glycol 6000, 2.5 M NaCl). The purified phages were used in the next round of selection and repeated binding, elution and infection steps for 2 times.

### Screening of clones by polyclonal phage ELISA

The screening was done with the entire population of phages eluted (fused to the antibody fragments) after each round of selection. ELISA plates (Nunc, Denmark) were coated with epsilon toxoid (100 µg/ml) overnight at 4 °C. The next day, plates were washed and blocked by 4% MPBS buffer per well. After washing, 10 µl of PEG precipitated phages recovered from each round of selection were diluted in 100 µl MPBS buffer and added to each well. One hour later, plates were washed and the binding of phages were detected using a monoclonal anti-c-Myc antibody (Biolegend) and anti-mouse HRP conjugate (Sigma Aldrich, USA) and detected with TMB substrate (Biobasic, Canada). Sulfuric acid solution 1 M was used to stop the reaction and enzymatic activity of HRP–antibody conjugate and the absorbance was read at 450 nm and 620 nm with a microplate reader.

### Screening of clones by monoclonal phage ELISA

For monoclonal phage ELISA, individual colonies were picked after the second and third rounds of selection and grown into 2xTY medium containing 100 µg/ml ampicillin and 4% glucose in a 96-well plate and incubated at 37 °C, 250 rpm for 12 hours. Then, the overnight culture for each clone diluted 100-fold into 200 µl 2xTY medium (100 µg/ml ampicillin and 0.1% glucose) and incubated at 37 °C, 250 rpm until OD600 = 0.4. The culture was infected with 4 × 10^8^ KM13 helper phages for 30 min at 37 °C, the bacteria pelleted by centrifugation and resuspended in 150 µl of 2 × YT containing ampicillin (100 µg/ml) and kanamycin (50 µg/ml) before growth overnight at 25 °C, 250 rpm for 16 hours. Then, the plate was centrifuged and 100 µl of culture supernatant of each well was used for ELISA plates that pre-coated with epsilon toxoid (100 µg/ml) and blocked with 4% MPBS buffer. Phage binding was detected with a monoclonal anti-c-Myc antibody (Biolegend) and anti-mouse HRP conjugate (Sigma).

### Screening of clones by scFv ELISA

Antibodies fused to the pIII coat protein of phage were converted to soluble scFv proteins by induction of bacteria with IPTG. scFv ELISAs were done similar to monoclonal phage ELISA. Soluble scFvs were produced by induction of culture of individual bacterial colonies at absorbance of about 0.9 at OD of 600 nm with 1 mM of IPTG and growth for 16 hour at 25 °C, 250 rpm in 2 × YT. For ELISA, the culture supernatants that contained soluble scFvs were transferred to the antigen-coated wells of ELISA plates. Bound scFvs were detected using 1:5000 monoclonal anti-Polyhistidine HRP conjugate (Sigma Aldrich, USA).

### Expression and purification of scFvs

The best clone of scFv ELISA from Tomlinson I + J libraries (Clone No. G_2_) was tested to confirm the presence of full-length insert by double digestion.

To improve the expression of anti-ETX scFv, the gene of selected clone (G_2_) was subcloned between the *Nco*I and *Not*I restriction sites of the pET26b (+) vector. The pET26b (+) vector contains the kanamycin resistance marker and carries an N-terminal pelB signal sequence for potential periplasmic localization in addition to an optional C-terminal His Tag sequence. CaCl_2_-treated *E. coli* BL21 (DE3) was used as the host strain and transformation was performed using the heat shock method. G_2_ scFv antibody was expressed using 0.01 mM IPTG for 24 hour at 25 °C. To extract the antibody, one gram of pelleted cell was resuspended in 5 ml ice-cold protein extraction buffer (50 mM Tris-HCl, 1 mM EDTA, 0.1% Triton X_100_ and 300 µg⁄ml lysozyme, pH 7.8) and was incubated for 30 min on ice. Then, the sample was sonicated at 24–25% amplitude for 6 sec ON and 6 sec OFF cycle on ice and total sonication time was 10 min. Following centrifugation at 10,000 g for 10 minutes at 4 °C supernatant containing total protein was mixed with 500 mM NaCl and 20 mM imidazole to purify the scFv protein by Ni-NTA affinity chromatography. Samples were loaded at a speed of 1 ml ⁄min onto the affinity column that had previously been equilibrated with cold binding buffer (50 mM Tris–HCl, 500 mM NaCl and 20 mM imidazole, pH 7.4). Unrelated bacterial proteins were eluted with binding buffer containing 50 mM imidazole before scFv recovery with binding buffer containing 400 mM imidazole. The purified protein fractions containing anti-ETX scFv were analyzed by SDS-PAGE and western blotting.

### Development a sandwich ELISA for quantitation of ETX

The sandwich ELISA was designed using B_1_ (the best clone isolated from DAb library) phage antibody as a capture antibody and soluble G_2_ scFv (the best clone isolated from Tomlinson I + J libraries) as a detection antibody. The optimal concentrations of capture (100 µg/ml to 0.005 µg/ml) and detection (100 µg/ml to 0.005 µg/ml) antibody were determined by checkerboard titration. To obtain a standard curve of the sandwich ELISA, microtiter plates were coated with 100 µl (25 µg/ml) capture phage VH antibody (B_1_) for overnight at 4 °C. After washing and blocking with 3% BSA/PBS (3% BSA in PBS), the 100 µl 10-fold serial dilutions of pure *C. perfringens* epsilon toxoid antigen from 5 to 50,000 ng/ml were added into the wells and incubated at 37 °C for 1 hour. The wells were washed five times with PBST (0.1% Tween-20 in PBS) buffer and once with PBS buffer and then 100 µl (50 µg/ml) G_2_ scFv antibody was added into the wells and incubated at 37 °C for 1 hour. Subsequently, 1:5000 dilution of HRP-conjugated monoclonal anti-Polyhistidine antibody as the conjugate antibody was added into all wells after washing. Subsequent addition of TMB substrate, the OD value was measured at 450 nm. The standard curve of the sandwich ELISA, were calculated using GraphPad Prism 8 software and 4- parameter logistic curve (4pl) to fit the standard curve. The limits of detection (LOD), limits of quantification (LOQ) were calculated based on the standard deviation of the blank (n = 6) as follows [25]:

$$ \mathrm{LOD}=\mathrm{Mean}\ \left(\mathrm{Blank}\right)+3.3\times \mathrm{Stdev}\kern0.5em \mathrm{blank}\kern0.5em \left(\mathrm{one}\kern0.5em \mathrm{sided}\kern0.5em 95\%\times 2\right) $$

$$ \mathrm{LOQ}=\mathrm{Mean}\ \mathrm{blank}+10\times \kern0.5em \mathrm{Stdev}\kern0.5em \mathrm{blank}\kern0.5em \left(\mathrm{one}\kern0.5em \mathrm{sided}\kern0.5em 95\%\times 6\right) $$

The designed ETX sandwich ELISA test was used to determine the concentration of ETX in an inactivated enterotoxemia commercial vaccine (RVSRI).

## Data Availability

All data generated or analyzed during this study are included in this published article and supplementary information files.

## Abbreviations

CDC: Centers for Disease Control and Prevention
ELISA: Enzyme-Linked Immunosorbent Assay
ETX: Epsilon toxin
DAb: Human domain antibody
PEG: Polyethylene glycol
RVSRI: Razi Vaccine and Serum Research Institute
scFv: Single-chain antibody fragment
